# Prognostic Factors and Impact of Therapeutic Intervention in Patients With Brain Metastases at the Initial Presentation

**DOI:** 10.7759/cureus.60368

**Published:** 2024-05-15

**Authors:** Yojiro Ishikawa, Satoshi Teramura, Hiroshi Nakano, Kengo Ito, Takayuki Yamada

**Affiliations:** 1 Division of Radiology, Tohoku Medical and Pharmaceutical University, Sendai, JPN; 2 Department of Radiation Oncology, Graduate School of Medicine, Tohoku University, Sendai, JPN

**Keywords:** brain and lung metastasis, brain, brain metasitasis, isolated brain metastasis, brain metastasis

## Abstract

Background

Studies investigating the normative characteristics and prognosis of patients diagnosed with brain metastases (BMs) at the onset of cancer are scarce. Therefore, we analyzed real-world treatment options.

Methodology

This retrospective study enrolled 112 patients newly diagnosed with BM between May 2006 and October 2021. The variables examined included patients’ age, sex, recurrence split analysis, Glasgow prognostic score (GPS), number of lesions, tumor size, peripheral brain tumor edema, targeted therapy, supportive care, chemotherapy, and date of onset. Prognostic factors were assessed using recursive partitioning analysis (RPA), graded prognostic assessment (GPA) scores, and GPS scoring, with magnetic resonance imaging (MRI) and computed tomography (CT) studies. Primary treatment comprised whole-brain radiotherapy (WBRT), with regular follow-up.

Results

Data from 112 survivors were analyzed, revealing a median overall survival time (MST) of 7.7 months, with some patients surviving beyond 24 months post-WBRT. Univariate analysis revealed associations between MST and RPA class, GPS, and treatment modalities (including targeted therapy and chemotherapy). RPA class 2, GPS of 0, and targeted therapy were identified as predictors of better prognosis in the multivariate analysis. In the subgroup not receiving chemotherapy, no significant difference in prognosis was seen between groups with or without WBRT.

Conclusions

Alongside RPA, scores indicating chronic inflammatory changes, including GPS, were confirmed as crucial prognostic factors. Moreover, treatment with molecularly targeted drugs correlated with favorable prognoses. The treatment-naïve group exhibited poorer prognoses, and WBRT was not deemed a significant prognostic factor in the chemotherapy group.

## Introduction

Brain metastasis (BM) represents a palliative manifestation that significantly impacts prognosis and quality of life (QOL) [1]. Effective management of BM is paramount for cancer patients, as 10%-40% of cancer patients eventually develop BM [2,3]. The diagnostic patterns of BM vary widely, including radiographic screenings at cancer diagnosis and staging, as well as follow-up observations in patients previously diagnosed with cancer [4]. Neuropathy and/or cognitive impairment may serve as initial clinical indicators of intracranial tumors, including distant metastases from extracranial primary tumors [5].

In previous reports of radiation therapy (RT), comparisons have been made across various modalities, such as whole-brain radiotherapy (WBRT), stereotactic radiosurgery (SRS), and combined surgical interventions [6]. However, the treatments available are often limited by each facility's capabilities. Some patients might not be referred due to limited medical resources, potentially resulting in scenarios where they receive only the best supportive care (BSC) or chemotherapy. RT may not always be essential for patients with poor prognoses [7]. Considering these points, data from analyses including not only RT cases but also those managed solely with chemotherapy or palliative treatment would be valuable to clinicians.

The prevalence of patients presenting with BM at the time of initial diagnosis ranges from 0.1% to 1.7% [8]. Although such cases are relatively rare clinically, patients with BM at initial diagnosis exhibit significantly poorer prognoses than those with subsequent diagnoses, indicated by the results of recursive partitioning analysis (RPA) and graded prognostic assessment (GPA) scores [9,10]. For instance, when BM is identified at initial diagnosis, RPA tends to classify cases as class ≥2 [11], with low GPA scores. Although some reports suggest relatively favorable prognoses for patients with BM at initial presentation, this area still requires further investigation, particularly regarding interventions for poor prognoses [12-14].

Collecting a homogeneous sample of BM cases is challenging due to the potential influences of previous treatment history. Conversely, patients diagnosed with BM at initial presentation or diagnosis are less likely to be affected by prior cancer treatment history. Furthermore, retrospective studies remain an important research modality due to the challenges in conducting randomized trials in the field of palliative care. Given the limited number of studies reporting on the baseline characteristics and prognosis of patients with BM at the time of their initial cancer diagnosis, we re-evaluated the treatment approach for these patients and conducted a retrospective analysis of the outcomes.

## Materials and methods

Eligibility criteria

This retrospective study was carried out in compliance with the guidelines stipulated in the Declaration of Helsinki, and the institutional review board approved this study (2024-2-003). Between May 2006 and October 2021, 112 patients diagnosed with BM at the time of their initial diagnosis were included in the study. The electronic medical records of patients with BMs in our institution were investigated. The surveyed items included the patients’ age (<65 or ≥65 years), sex (male or female), RPA (class 2 or class 3) class, Glasgow prognostic score (0 or 1-2), number of lesions (≤3 or >3, ≤5 or >5, ≤10 or >10), tumor size (≤2.0 or >2.0 cm), peripheral brain tumor edema (present or absent), targeted therapy (with or without), BSC (yes or no), chemotherapy (with or without), and date of onset (2008-2014 or 2015-2021).

Prognosis factor scoring

RPA was defined as age, Karnofsky Performance Score (KPS), and extracranial lesion status. Patients were classified as having RPA class 2 or 3 because their primary tumor was uncontrolled, except for patients with cancer of unknown primary [11]. GPA was defined as age, KPS, extracranial lesion status, and number of BMs, as previously published [9,10]. GPS was defined as the serum albumin and C-reaction protein levels. Patients with both an elevated C-reactive protein level (>10 mg/L) and hypoalbuminemia (<35 g/L) were assigned a GPS of 2. Patients in whom only one of those biochemical abnormalities was present were assigned a GPS of 1. Patients in whom neither of those abnormalities was present were assigned a GPS of 0 [15-17].

Imaging

Gadolinium-enhanced MRI was conducted for patients, whereas MRI without contrast or CT was utilized in resource-limited settings or for patients with contraindications to MRI. MRI with a 1.5-T MRI system included axial T1-weighted spin-echo, axial T2-weighted spin-echo, and axial gadolinium-enhanced T1-weighted spin-echo sequences. Additional sequences, including fluid attenuated inversion recovery and susceptibility weighted imaging, were employed for certain patients. The image analysis in this study was performed by one experienced radiologist and one neuroradiation oncologist.

WBRT

All three-dimensional conformal radiation therapy (3DCRT)-treated patients were immobilized in the supine position. CT was performed at a slice thickness of 2.5 mm with a multidetector CT scanner (GE LightSpeed QX/i; GE Healthcare, Waukesha, WI). The Eclips treatment planning system (Varian Medical Systems, Palo Alto, CA) was used for dose calculations during 3DCRT planning.

Outcome assessments and follow-up

OS was defined as the period from the date of confirmed diagnosis for BSC, the date of RT initiation for RT cases, and the date of systemic chemotherapy initiation for chemotherapy cases, to the date of death from any cause. Follow-up assessments were conducted at the attending physician's discretion and included blood sampling, CT of the trunk, and MRI or CT of the head every one to three months. These assessments were scheduled every one to three months during the first year and every six months thereafter.

Statistical analysis

Statistical analyses were performed using JMP® v16 (SAS Institute Inc., Cary, NC). Statistical significance was set at *P* < 0.05 in all analyses. The OS rates were determined using Kaplan-Meier estimates. The clinical parameters were investigated using univariate analysis (log-rank test). Hazard ratios with 95% confidence intervals (95% CIs) were calculated by using Cox’s proportional hazard regression model with the following variables: (1) RPA class 2, (2) GPS = 0, (3) GPS of 0-1, (4) with targeted therapy, and (5) BSC.

In the subgroup analysis, the difference in prognosis between the patients with and without WBRT who did not receive chemotherapy, as well as the patients’ age (<65 or >65 years), sex (male or female), recurrence split analysis (class II or III), Glasgow prognostic score (0 or 1-2), number of lesions (≤3 or >3, ≤5 or >5, ≤10 or >10), tumor diameter (≤2.0 or >2.0 cm), peripheral brain tumor edema (yes or no), targeted therapy (yes or no), BSC (yes or no), chemotherapy (yes or no), and onset date (2008-2014 or 2015-2021) were analyzed.

## Results

Patient and brain tumor characteristics

Data obtained from all 112 patients were included in the survival analysis. Table 1 shows the patient and tumor characteristics. Altogether, 335 cases of BM were detected in the countable range (median: 2 per patient). The BM localization varied, with 50 patients having lesions localized to the cerebrum, 10 to the brainstem, and 20 to the cerebellum. Three patients had metastases to the hippocampus and hippocampal region, and two patients had metastases to the amygdala.

**Table 1:** Patient and tumor characteristics.

	*N* or range	% or median
No. of patients	112	100%
Age (years)	38-91 years	Median 69 years
Male/Female	76/36	67.9%/32.1%
Karnofsky Performance Status		
70-100	47	42.0%
50-60	41	36.6%
0-40	18	16.0%
Missing	6	5.4%
Recursive partitioning analysis		
Class 2	94	83.9%
Class 3	18	16.1%
Glasgow prognostic score		
0	59	52.7%
1	27	24.1%
2	22	19.6%
Missing	4	3.6%
Glasgow prognostic score (Modified)		
0	46	41.1%
1	36	32.1%
2	26	23.2%
Missing	4	3.6%
Primary lesion site		
Lung	96	85.7%
Gastrointestinal	6	5.4%
Breast	3	2.7%
Esophageal	1	0.9%
Rectal	1	0.9%
Skin	1	0.9%
Unknown	4	3.5%
Histology		
Adenocarcinoma	50	44.7%
Squamous cell carcinoma	10	8.9%
Small-cell carcinoma	19	16.9%
Sarcoma	1	0.9%
Other	14	12.5%
Unclear	18	16.1%
Number of lesions	1-31 lesions	median 2 lesions
≤3 lesions	62	55.4%
>3 lesions	44	39.3%
Other	6	5.3%
>10 lesions	21	18.8%
≤10 lesions	85	75.9%
Other	6	5.3
Tumor size (cm)	0.3–5.0cm	median 1.6 cm
Whole brain radiation therapy		
With	48	42.9%
Without	64	57.1%
Chemotherapy		
With	58	51.8%
Without	54	48.2%
Targeted therapy		
With	19	16.9%
Without	93	83.1%
Best supportive care		
Yes	41	63.4%
No	71	36.6%
Brain surgery		
With	2	1.8%
Without	110	98.2%
Stereotactic radiation therapy		
With	2	1.8%
Without	110	98.2%

Survival

**Figure 1:**
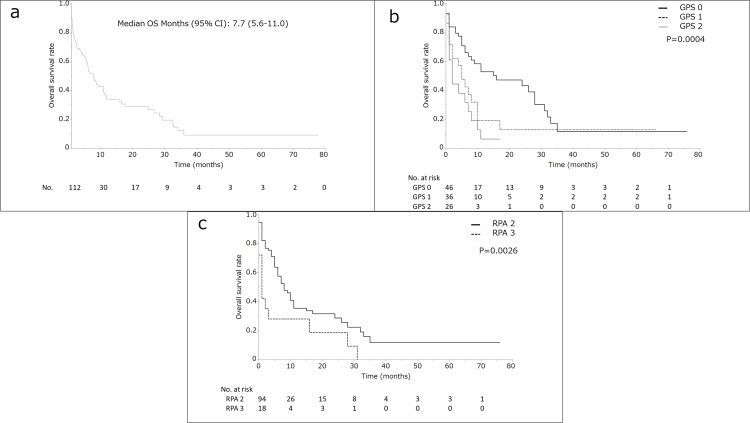
The overall survival rate of patients with brain metastases at the initial presentation and comparative overall survival results. The overall survival (OS) rate of patients with brain metastases at the initial presentation (*N* = 112) (a). Comparative overall survival results, recursive partitioning analysis (RPA): classes 2 and 3 (b). Comparative OS results, Glasgow prognostic score (GPS) groups: 0, 1, and 2 (c).

The median overall survival time (MST) was 7.7 months (95% CI 5.6-11.0 months) (Figure 1a). Of note, 17 patients (9.4%) survived for >24 months after WBRT, with MST of 36 months (range 24-37 months), 16 patients had fewer than five BMs, and the median WBRT dose was 30 Gy (range 20-36 Gy). During this prognostic study, 68 patients had died. Of the 37 patients who survived, 15 were transferred to palliative care units to receive BSC. Three patients were long-term survivors. One had breast cancer, and two had lung cancer with one to two BMs. One lung cancer patient was on molecular-targeted drugs, and two were aged <50 years.

Univariate and multivariate outcomes

In the univariate analysis, a GPS  of 0 was significantly associated with increased MST (15.8, 95% CI 6.5-29.2, vs. 4.4, 95% CI 2.0-7.7 months, *P* = 0.0005). A GPS of 0-1 increased the MST (9.4, 95% CI 6.1-16.6, vs. 1.4, 95% CI:1.4-6.1 months, *P* < 0.0001]) (Figure 1b). Patients with RPA classes 2 and 3 had MST of 8.7 (95% CI 6.5-11.8) and 1.7 (95% CI 0.6-3.9) months, respectively (*P* = 0.026) (Figure 1c). 

**Figure 2:**
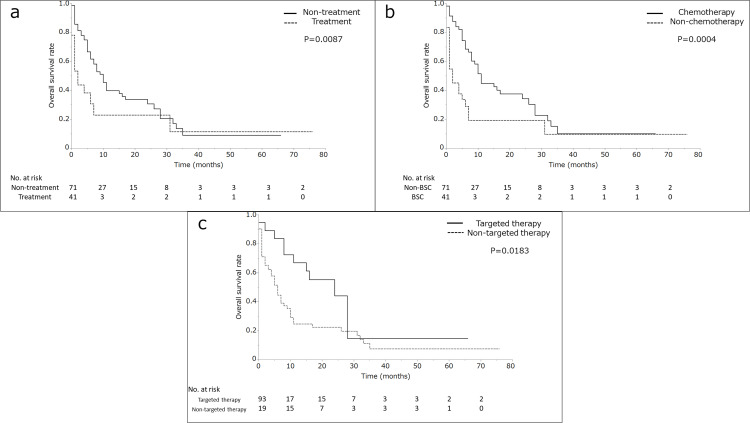
Comparative overall survival results. Comparative overall survival (OS) results, therapeutic intervention: nontreatment (best supportive care) and treatment (chemotherapy, targeted therapy, surgery, or radiation therapy) (a). Comparative OS results, treatment with and without chemotherapy (b). Comparative OS results, treatment with and without targeted therapy (c).

A significant difference was found in MST between the patients receiving and not receiving treatment (BSC) (10.8, 95% CI 7.6-15.8, vs. 1.2, 95% CI 1.2-7.7 months, *P* = 0.087) (Figure 2a). The patients receiving chemotherapy had increased MST (11.4, 95% CI 8.6-17.6, vs. 2.2, 95% CI 1.4-5.6 months, *P* = 0.0004) (Figure 2b). The patients receiving targeted therapy had also increased MST (24.9, 95% CI 8.7-29.2, vs. 6.1, 95% CI 3.9-8.0 months, *P* = 0.015) (Figure 2c).

**Figure 3:**
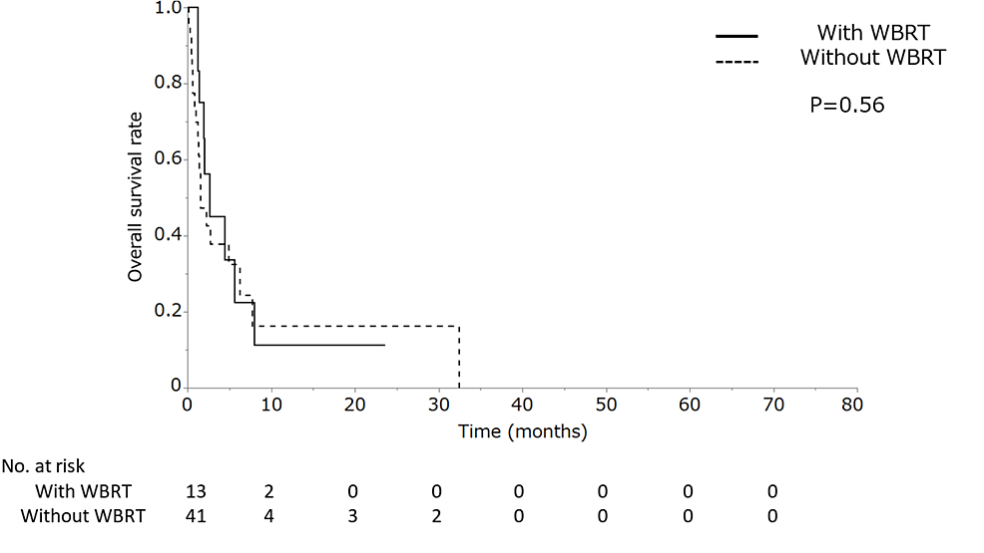
Comparative overall survival results in the subgroup without chemotherapy. Comparative overall survival results in the subgroup without chemotherapy, those receiving whole-brain radiation therapy (WBRT), and those without WBRT.

Univariate analysis revealed that none of the following variables were significant prognostic factors: age, sex, number of lesions, tumor size, peripheral brain tumor edema, WBRT, and date of onset. Multivariate analysis showed that RPA class 2, GPS of  0, and targeted therapy significantly indicated a good prognosis (Table 2). The Glasgow prognostic score (0 or 1-2) predominantly prolonged MST. The subgroup analysis showed no difference in prognosis in patients with and without WBRT not receiving chemotherapy (Figure 3).

**Table 2:** Univariate and multivariate analyses of prognostic parameters after whole-brain radiation therapy.

Parameter	Univariate		Multivariate	
	Median OS months (95% CI)	*P*-value	HR (95% CI)	*P*-value
Age (years)				
<65	11.4 (6.2-24.9)	0.092		
≥65	7.6 (3.5-10.8)			
Sex				
Male	7.7 (5.6-10.8)	0.19		
Female	8.7 (4.9-32.7)			
Recursive partitioning analysis				
Class 2	8.7 (6.5-11.8)	0.0026	0.45 (0.26-0.9)	0.026
Class 3	1.7 (0.6-3.9)			
Glasgow prognostic score				
0	15.8 (6.5-29.2)	0.0006	0.38 (0.2-0.9)	0.0029
1-2	4.4 (2.0-7.7)			
0-1	9.4 (6.1-16.6)	<0.0001	0.75 (0.37-1.54)	0.44
2	1.4 (1.4-6.1)			
Number of lesions				
≤3	8.7 (6.2-11.9)	0.21		
>3	6.2 (3.9-11.8)			
≤5	8.7 (6.2-11.9)	0.16		
>5	6.0 (3.5-11.8)			
≤10	8.0 (5.9-11.4)	0.85		
>10	6.2 (2.2-29.2)			
Tumor size (cm)				
≤2.0	8.7 (5.7-15.8)	0.54		
>2.0	7.7 (4.0-17.6)			
Edema peripheral brain tumors: Present/Absent	5.7 (2.4-10.3)/9.4 (7.6-11.8)	0.42		
Targeted therapy				
With	24.9 (8.7-29.2)	0.015	0.46 (0.22-0.96)	0.037
Without	6.1 (3.9-8.0)			
Best supportive care				
Yes	1.2 (1.2-7.7)	0.0087	1.1 (0.48-2.58)	0.81
No	10.8 (7.6-15.8)			
Chemotherapy				
With	11.4 (8.6-17.6)	0.0004	0.54 (0.24-1.2)	0.13
Without	2.2 (1.4-5.6)			
Date of onset				
2008-2014	7.6 (4.4-16.6)	0.29		
2015-2021	8.7 (4.4-16.6)			

## Discussion

Metastasis remains one of the primary causes of mortality in cancer patients [18], with patients diagnosed with BM facing a grim prognosis. According to RPA, the survival rates for patients with BM ranged from 2.3 to 7.1 months, varying based on disease severity [11]. Although the present study focuses on a unique subset of cases involving BMs diagnosed at the initial presentation, the median survival observed was slightly longer than the RPA estimates. Cases with BMs at the time of initial diagnosis might have a more favorable prognosis. The marginally extended prognosis observed in this study could be attributed to the inclusion of a significant number of patients with access to potential interventions in the future. Notably, the study revealed a longer prognosis in the group receiving chemotherapy and molecular-targeted therapy, which contrasts with the shorter prognosis in the group receiving BSC. The primary tumor type may also influence the outcomes. In diagnosis-specific graded prognostic assessment (DS-GPA), which considers primary tumor type and characteristics, the survival ranged from 2.79 to 25.30 months [19], similar to the results of this study. However, given that the lung cancer patients constituted 85% of the cases, it is plausible that this represented a series of cases with relatively short prognoses. Previous reports indicating a life expectancy of approximately one month without intervention align with the prognosis observed in the BSC group in this case series [20].

Nowadays, SRS is established as the first-line treatment for localized BMs, suggesting no survival benefit to justify the detrimental impact of WBRT on cognitive function and QOL in cancer patients with BMs [21-25]. The results of a phase III randomized clinical trial comparing SRS with and without WBRT for BMs indicate that despite the superior central nervous system control provided by WBRT, adding WBRT to SRS does not provide an overall survival (OS) advantage, which has been shown to be unobtainable [6]. Given these results, WBRT has been less aggressively performed in recent years. However, for patients with BMs at initial presentation, such as those in this study, SRS was rarely chosen, although the median number of BMs was approximately two. Since our facility cannot perform SRS, many of the patients in this study had poor performance status (PS), making it challenging to refer them to facilities that actively provide SRS. In facilities like ours, which lack the equipment needed to perform SRS, the choice of SRS for patients with BMs at initial presentation may not be prevalent among clinicians treating cancer in real-world conditions, even for a small number of cases with BMs.

WBRT poses complications to cognitive function in cancer patients with BMs [21-25]. WBRT that spares the hippocampus, which affects cognitive function, through the use of intensity-modulated radiation therapy and other modalities, has become the treatment of choice [26]. However, some clinical scenarios still require classical 3DCRT, such as in cases with a short prognosis where the primary tumor or intracranial lesions have become uncontrollable due to lack of response to chemotherapy [27], or when the neurological findings due to BMs are urgent. One of the significant findings of this study is that WBRT using older 3DCRT technology remains a viable option for treating BMs at the initial diagnosis. Furthermore, patients with small-cell lung cancer (SCLC) have been excluded from the landmark randomized clinical trials that established SRS alone as the first-line strategy, making SCLC an exception where WBRT remains the standard of care for limited and even isolated BMs [28]. Nevertheless, there is growing interest in the potential role of SRS in BM treatment, even for SCLC [29,30]. Although treatment options for BMs from SCLC may ultimately depend on each facility's policies, SCLC cases are included in 16.9% of this study. In instances of BMs from SCLC at initial diagnosis, WBRT can be considered a treatment option.

The following prognostic factors for BMs have been proposed: KPS, RPA, and GPA, which are some of those recognized in the Radiation Therapy Oncology Group studies [11,31]. In the present study, all patients in the poor prognosis group were considered high risk because they had BMs at the time of initial diagnosis. There was also a trend toward a greater number of patients with lung cancer. DS-GPA has been proposed for lung, breast, gastrointestinal, and renal cancers as well as malignant melanoma [32]. Recently, the prognosis of lung cancer has been dependent on the presence of EGFR mutations and ALK fusion protein [33]. In our study, 96 patients (85.7%) had lung cancer, including 37 patients with lung adenocarcinoma. Targeted therapy was used in 29 patients (16.1%), but it was unclear in our study whether patients with BMs at the initial presentation benefited from the recent targeted therapies.

Determining whether extracranial lesions can be controlled in patients with BMs at initial presentation requires a high degree of clinical judgment, but if the results of this study can be used to identify patients who can be treated with chemotherapy or targeted therapy, it will contribute to the therapeutic strategy of RT. GPS is a prognostic score that evaluates the inflammatory changes that have recently been proposed for various diseases. It is considered to be one of the prognostic factors for patients with RPA class ≥2. In the present study, GPS was calculated as a prognostic factor in a multivariate analysis, and may be useful for patients for whom PS is difficult to evaluate or as an aid in PS evaluation. Furthermore, even if a patient has a single metastasis, some cases may require whole-brain irradiation or BSC without whole-brain irradiation, depending on the progression of the primary tumor. Although this remains an unresolved issue, the GPS score, which reflects systemic inflammation, can be used as an indicator for determining whether whole-brain irradiation should be omitted in patients with a short prognosis. Although it is difficult to strongly mention in this study, WBRT with hippocampal preservation may be an option in patients with a good prognosis who can use chemotherapy or molecular-targeted therapies for BMs at the initial diagnosis. However, the frequency of hippocampal metastases in this study was very low, and it is unclear whether hippocampus-preserving irradiation is recommended in patients with BMs at the initial diagnosis.

Various irradiation doses have been used in WBRT treatment strategies, including 20 Gy/4-5 fractions, 30 Gy/15 fractions, 30 Gy/10 fractions, 37.5 Gy/15 fractions, and 40 Gy/20 fractions [34]. In our study, there was a relatively minimal variation in whole-brain irradiation, 20-36 Gy, and there was likely no prognostic effect of WBRT as compared to BSC. The 40 Gy/20 fractions was found to be more effective in controlling intracranial tumors in two randomized controlled trials as compared to the 40 Gy/20 fractions, which was found to be significantly superior to 20 Gy/4-5 fractions in controlling the intracranial tumors in two randomized controlled trials [31,35,36]. In addition, increased doses are an option for patients with a longer prognosis. Good outcomes have also been reported in patients receiving 2 to 2.5 Gy of WBRT for breast cancer [37]. In the present study, very few patients with breast cancer (*N* = 2) may have shown an impact on prognosis.

The present study had several limitations. Bias in patient selection for the analysis of OS rates cannot be ruled out, as this study was retrospective in nature and only included patients who underwent WBRT. Furthermore, as only OS was evaluated in this study, it may represent the prognosis of patients eligible for chemotherapy or WBRT rather than the efficacy of chemotherapy or WBRT. The prognosis was short, and assessing the tumor response to treatment modalities was difficult because some patients did not undergo follow-up imaging. Moreover, the fact that progression-free survival was not assessed was an important drawback. Many patients were transferred to home treatment, palliative care hospitals, or hospices, and detailed information was unavailable. For RT, the total dose was left to the discretion of each attending physician. Given that QOL and cognitive function were not assessed before treatment, analyzing the progression of QOL and cognitive function with each treatment method was difficult.

## Conclusions

In conclusion, besides RPA, proposed as a prognostic factor, we identified a score indicative of chronic inflammatory changes, i.e., GPS, as a potential prognostic factor. The administration of molecular-targeted drugs was also linked with a significantly better prognosis. The prognosis for the treatment-naïve (BSC) group was notably poor, and WBRT did not emerge as a prognostic factor for the chemotherapy-naïve cohort.
